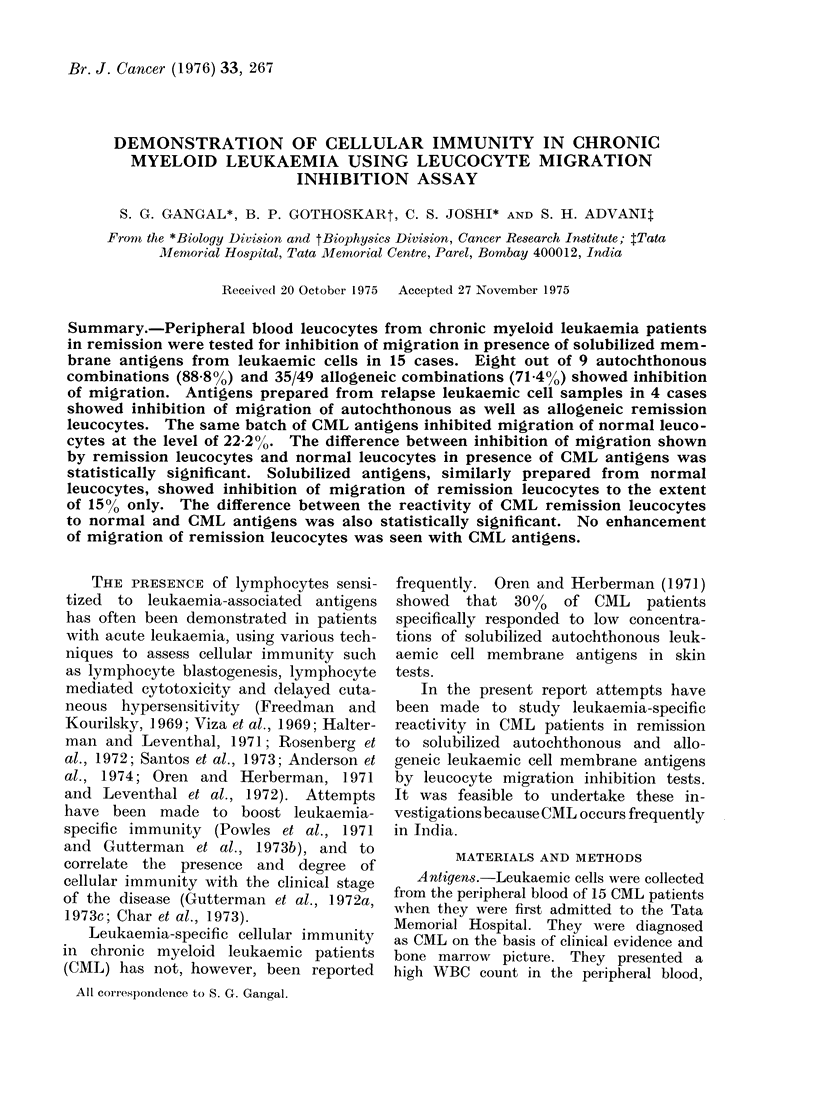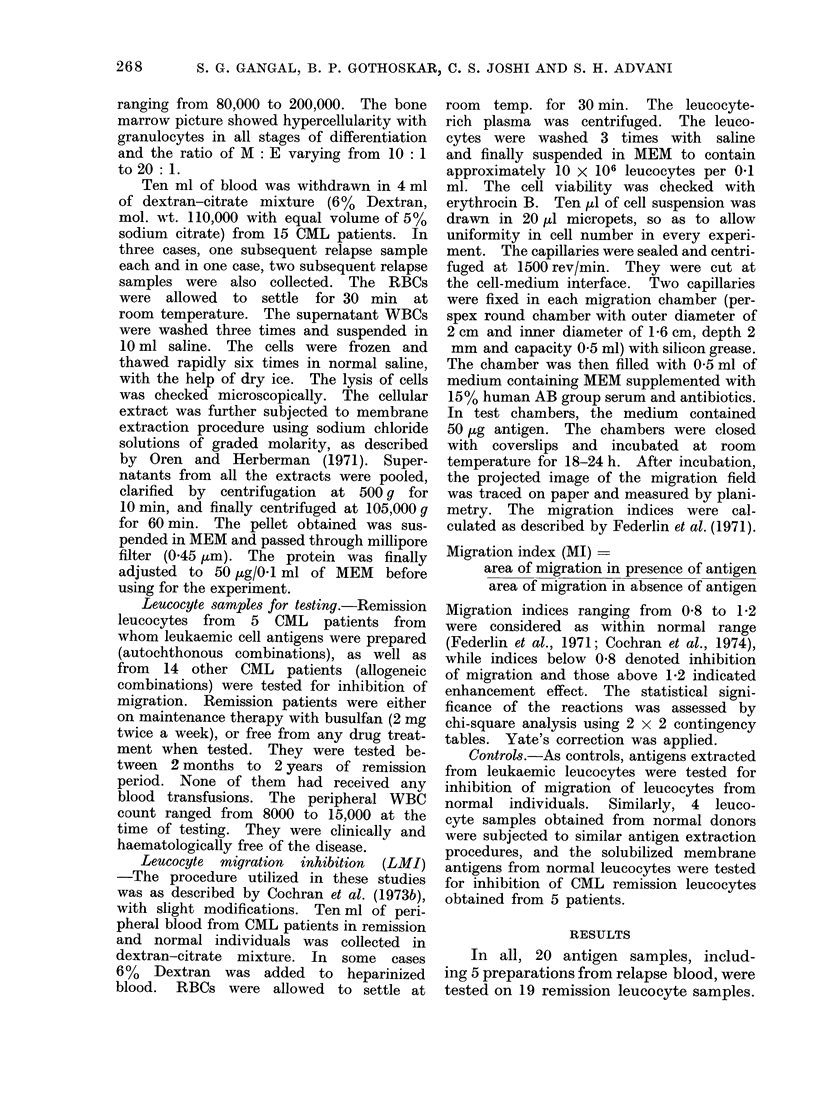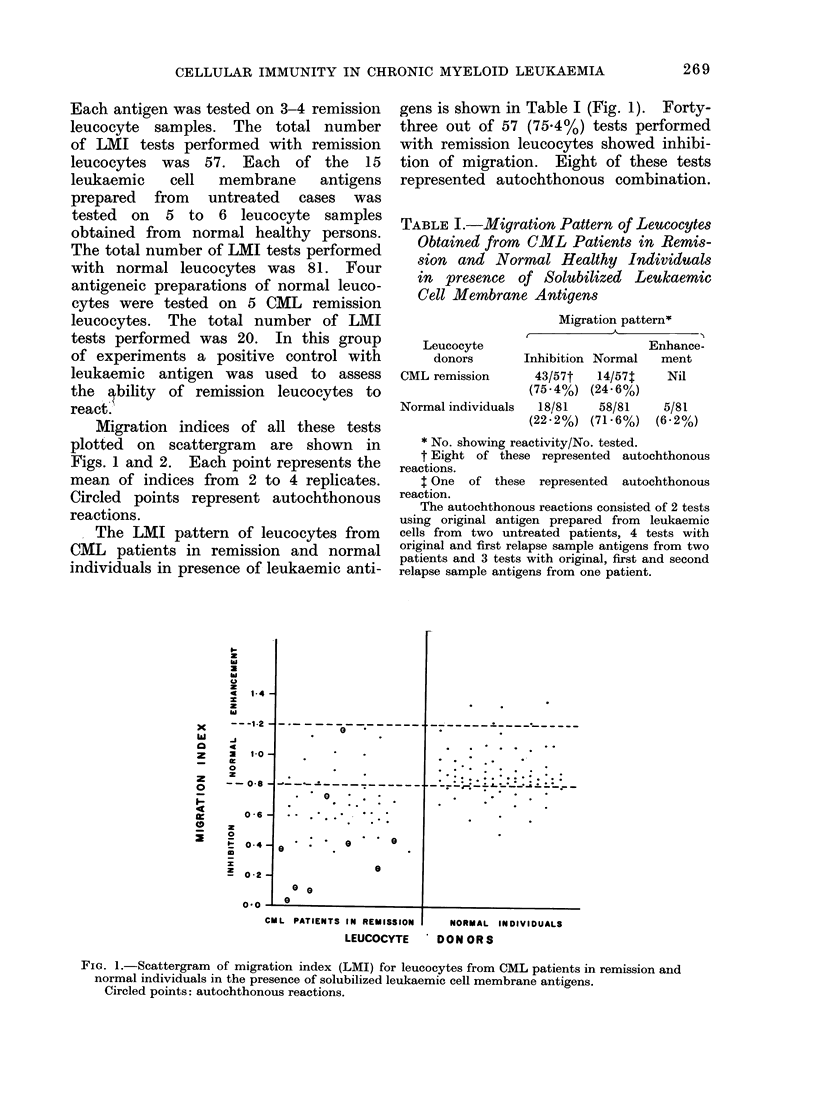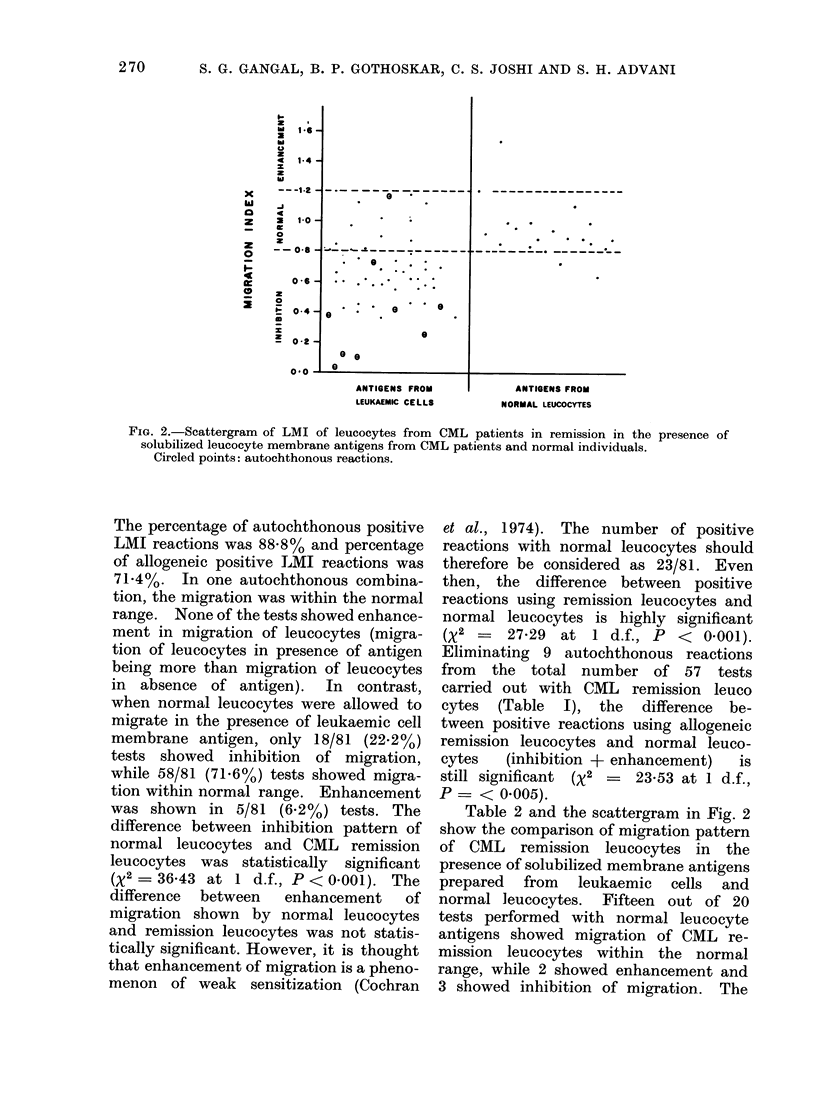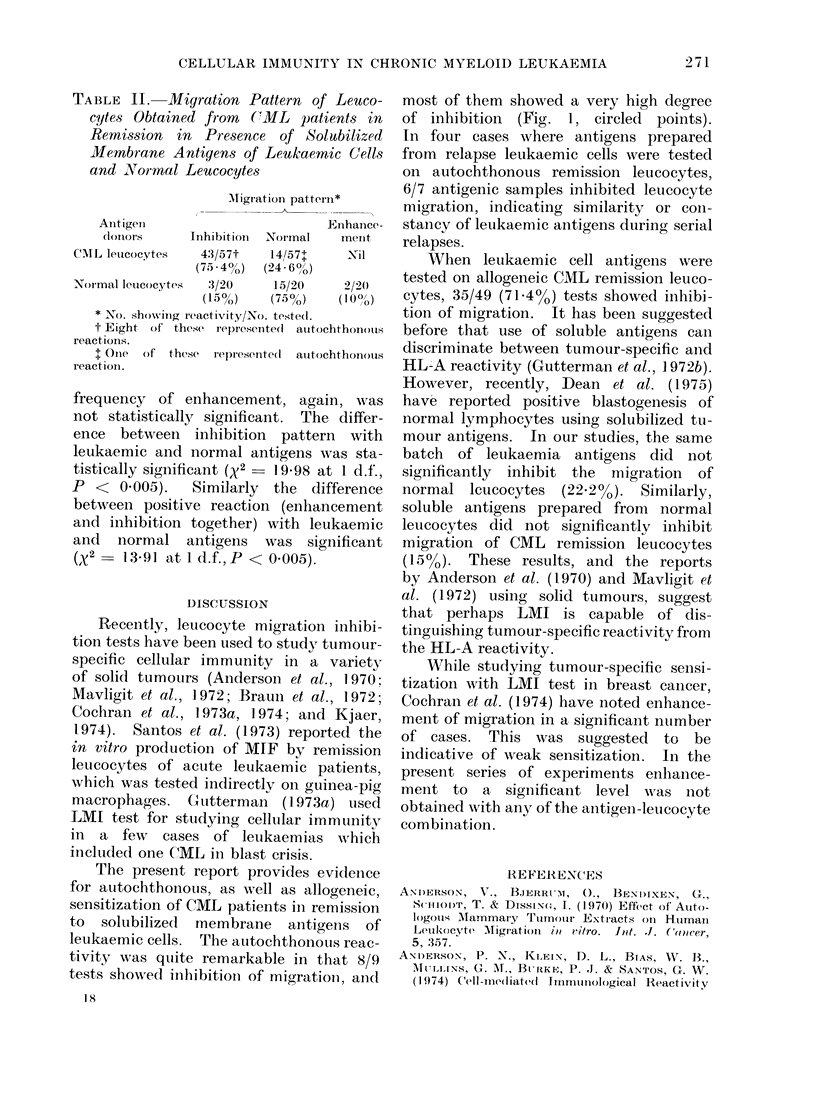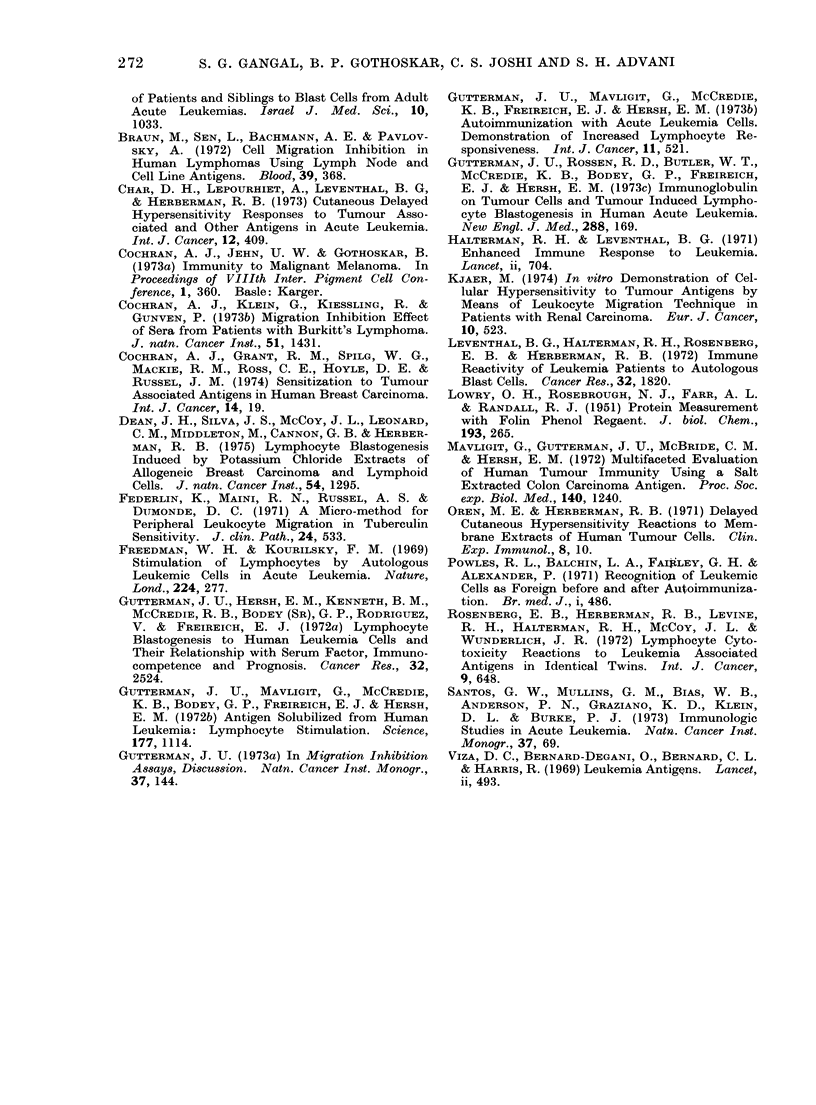# Demonstration of cellular immunity in chronic myeloid leukaemia using leucocyte migration inhibition assay.

**DOI:** 10.1038/bjc.1976.39

**Published:** 1976-03

**Authors:** S. G. Gangal, B. P. Gothoskar, C. S. Joshi, S. H. Advani

## Abstract

Peripheral blood leucocytes from chronic myeloid leukaemia patients in remission were tested for inhibition of migration in presence of solubilized membrane antigens from leukaemic cells in 15 cases. Eight out of 9 autochthonous combinations (88-8%) and 35/49 allogenic combinations (71-4%) showed inhibition of migration. Antigens prepared from relapse leukaemic cell samples in 4 cases showed inhibition of migration of autochthonous as well as allogeneic remission leucocytes. The same batch of CML antigens inhibited migration of normal leucocytes at the level of 22-2%. The difference between inhibition of migration shown by remission leucocytes and normal leucocytes in presence of CML antigens was statistically significant. Solubilized antigens, similarly prepared from normal leucocytes, showed inhibition of migration of remission leucocytes to the extent of 15% only. The difference between the reactivity of CML remission leucocytes to normal and CML antigens was also statistically significant. No enhancement of migration of remission leucocytes was seen with CML antigens.


Br. J. Cancer (1976) 33, 267

### DEMONSTRATION OF CELLULAR IMMUNITY IN CHRONIC MYELOID LEUKAEMIA USING LEUCOCYTE MIGRATION INHIBITION ASSAY

S. G. GANGAL*, B. P. GOTHOSKAR†, C. S. JOSHI* AND S. H. ADVANI‡

*From the *Biology Division and †Biophysics Division, Cancer Research Institute; ‡Tata Memorial Hospital, Tata Memorial Centre, Parel, Bombay 400012, India*

Received 20 October 1975 Accepted 27 November 1975

**Summary.—Peripheral blood leucocytes from chronic myeloid leukaemia patients in remission were tested for inhibition of migration in presence of solubilized membrane antigens from leukaemic cells in 15 cases. Eight out of 9 autochthonous combinations (88·8%) and 35/49 allogeneic combinations (71·4%) showed inhibition of migration. Antigens prepared from relapse leukaemic cell samples in 4 cases showed inhibition of migration of autochthonous as well as allogeneic remission leucocytes. The same batch of CML antigens inhibited migration of normal leucocytes at the level of 22·2%. The difference between inhibition of migration shown by remission leucocytes and normal leucocytes in presence of CML antigens was statistically significant. Solubilized antigens, similarly prepared from normal leucocytes, showed inhibition of migration of remission leucocytes to the extent of 15% only. The difference between the reactivity of CML remission leucocytes to normal and CML antigens was also statistically significant. No enhancement of migration of remission leucocytes was seen with CML antigens.**

THE PRESENCE of lymphocytes sensitized to leukaemia-associated antigens has often been demonstrated in patients with acute leukaemia, using various techniques to assess cellular immunity such as lymphocyte blastogenesis, lymphocyte mediated cytotoxicity and delayed cutaneous hypersensitivity (Freedman and Kourilsky, 1969; Viza *et al.*, 1969; Halterman and Leventhal, 1971; Rosenberg *et al.*, 1972; Santos *et al.*, 1973; Anderson *et al.*, 1974; Oren and Herberman, 1971 and Leventhal *et al.*, 1972). Attempts have been made to boost leukaemia-specific immunity (Powles *et al.*, 1971 and Gutterman *et al.*, 1973*b*), and to correlate the presence and degree of cellular immunity with the clinical stage of the disease (Gutterman *et al.*, 1972*a*, 1973*c*; Char *et al.*, 1973).

Leukaemia-specific cellular immunity in chronic myeloid leukaemic patients (CML) has not, however, been reported frequently. Oren and Herberman (1971) showed that 30% of CML patients specifically responded to low concentrations of solubilized autochthonous leukaemic cell membrane antigens in skin tests.

In the present report attempts have been made to study leukaemia-specific reactivity in CML patients in remission to solubilized autochthonous and allogeneic leukaemic cell membrane antigens by leucocyte migration inhibition tests. It was feasible to undertake these investigations because CML occurs frequently in India.

### MATERIALS AND METHODS

*Antigens.*—Leukaemic cells were collected from the peripheral blood of 15 CML patients when they were first admitted to the Tata Memorial Hospital. They were diagnosed as CML on the basis of clinical evidence and bone marrow picture. They presented a high WBC count in the peripheral blood,

All correspondence to S. G. Gangal.

ranging from 80,000 to 200,000. The bone marrow picture showed hypercellularity with granulocytes in all stages of differentiation and the ratio of M : E varying from 10 : 1 to 20 : 1.

Ten ml of blood was withdrawn in 4 ml of dextran–citrate mixture (6% Dextran, mol. wt. 110,000 with equal volume of 5% sodium citrate) from 15 CML patients. In three cases, one subsequent relapse sample each and in one case, two subsequent relapse samples were also collected. The RBCs were allowed to settle for 30 min at room temperature. The supernatant WBCs were washed three times and suspended in 10 ml saline. The cells were frozen and thawed rapidly six times in normal saline, with the help of dry ice. The lysis of cells was checked microscopically. The cellular extract was further subjected to membrane extraction procedure using sodium chloride solutions of graded molarity, as described by Oren and Herberman (1971). Supernatants from all the extracts were pooled, clarified by centrifugation at 500 *g* for 10 min, and finally centrifuged at 105,000 *g* for 60 min. The pellet obtained was suspended in MEM and passed through millipore filter (0·45 μm). The protein was finally adjusted to 50 μg/0·1 ml of MEM before using for the experiment.

*Leucocyte samples for testing.*—Remission leucocytes from 5 CML patients from whom leukaemic cell antigens were prepared (autochthonous combinations), as well as from 14 other CML patients (allogeneic combinations) were tested for inhibition of migration. Remission patients were either on maintenance therapy with busulfan (2 mg twice a week), or free from any drug treatment when tested. They were tested between 2 months to 2 years of remission period. None of them had received any blood transfusions. The peripheral WBC count ranged from 8000 to 15,000 at the time of testing. They were clinically and haematologically free of the disease.

*Leucocyte migration inhibition* (*LMI*)—The procedure utilized in these studies was as described by Cochran *et al.* (1973*b*), with slight modifications. Ten ml of peripheral blood from CML patients in remission and normal individuals was collected in dextran–citrate mixture. In some cases 6% Dextran was added to heparinized blood. RBCs were allowed to settle at room temp. for 30 min. The leucocyte-rich plasma was centrifuged. The leucocytes were washed 3 times with saline and finally suspended in MEM to contain approximately $10 \times 10^6$ leucocytes per 0·1 ml. The cell viability was checked with erythrocin B. Ten μl of cell suspension was drawn in 20 μl micropets, so as to allow uniformity in cell number in every experiment. The capillaries were sealed and centrifuged at 1500 rev/min. They were cut at the cell-medium interface. Two capillaries were fixed in each migration chamber (perspex round chamber with outer diameter of 2 cm and inner diameter of 1·6 cm, depth 2 mm and capacity 0·5 ml) with silicon grease. The chamber was then filled with 0·5 ml of medium containing MEM supplemented with 15% human AB group serum and antibiotics. In test chambers, the medium contained 50 μg antigen. The chambers were closed with coverslips and incubated at room temperature for 18–24 h. After incubation, the projected image of the migration field was traced on paper and measured by planimetry. The migration indices were calculated as described by Federlin *et al.* (1971).

$$\text{Migration index (MI)} = \frac{\text{area of migration in presence of antigen}}{\text{area of migration in absence of antigen}}$$

Migration indices ranging from 0·8 to 1·2 were considered as within normal range (Federlin *et al.*, 1971; Cochran *et al.*, 1974), while indices below 0·8 denoted inhibition of migration and those above 1·2 indicated enhancement effect. The statistical significance of the reactions was assessed by chi-square analysis using $2 \times 2$ contingency tables. Yate's correction was applied.

*Controls.*—As controls, antigens extracted from leukaemic leucocytes were tested for inhibition of migration of leucocytes from normal individuals. Similarly, 4 leucocyte samples obtained from normal donors were subjected to similar antigen extraction procedures, and the solubilized membrane antigens from normal leucocytes were tested for inhibition of CML remission leucocytes obtained from 5 patients.

### RESULTS

In all, 20 antigen samples, including 5 preparations from relapse blood, were tested on 19 remission leucocyte samples.

Each antigen was tested on 3–4 remission leucocyte samples. The total number of LMI tests performed with remission leucocytes was 57. Each of the 15 leukaemic cell membrane antigens prepared from untreated cases was tested on 5 to 6 leucocyte samples obtained from normal healthy persons. The total number of LMI tests performed with normal leucocytes was 81. Four antigeneic preparations of normal leucocytes were tested on 5 CML remission leucocytes. The total number of LMI tests performed was 20. In this group of experiments a positive control with leukaemic antigen was used to assess the ability of remission leucocytes to react.

Migration indices of all these tests plotted on scattergram are shown in Figs. 1 and 2. Each point represents the mean of indices from 2 to 4 replicates. Circled points represent autochthonous reactions.

The LMI pattern of leucocytes from CML patients in remission and normal individuals in presence of leukaemic antigens is shown in Table I (Fig. 1). Forty-three out of 57 (75·4%) tests performed with remission leucocytes showed inhibition of migration. Eight of these tests represented autochthonous combination.

TABLE I.—*Migration Pattern of Leucocytes Obtained from CML Patients in Remission and Normal Healthy Individuals in presence of Solubilized Leukaemic Cell Membrane Antigens*

| | Migration pattern* | | |
|---|---|---|---|
| Leucocyte donors | Inhibition | Normal | Enhancement |
| CML remission | 43/57† (75·4%) | 14/57‡ (24·6%) | Nil |
| Normal individuals | 18/81 (22·2%) | 58/81 (71·6%) | 5/81 (6·2%) |

* No. showing reactivity/No. tested.

† Eight of these represented autochthonous reactions.

‡ One of these represented autochthonous reaction.

The autochthonous reactions consisted of 2 tests using original antigen prepared from leukaemic cells from two untreated patients, 4 tests with original and first relapse sample antigens from two patients and 3 tests with original, first and second relapse sample antigens from one patient.

FIG. 1.—Scattergram of migration index (LMI) for leucocytes from CML patients in remission and normal individuals in the presence of solubilized leukaemic cell membrane antigens.
Circled points: autochthonous reactions.

FIG. 2.—Scattergram of LMI of leucocytes from CML patients in remission in the presence of solubilized leucocyte membrane antigens from CML patients and normal individuals.
Circled points: autochthonous reactions.

The percentage of autochthonous positive LMI reactions was 88·8% and percentage of allogeneic positive LMI reactions was 71·4%. In one autochthonous combination, the migration was within the normal range. None of the tests showed enhancement in migration of leucocytes (migration of leucocytes in presence of antigen being more than migration of leucocytes in absence of antigen). In contrast, when normal leucocytes were allowed to migrate in the presence of leukaemic cell membrane antigen, only 18/81 (22·2%) tests showed inhibition of migration, while 58/81 (71·6%) tests showed migration within normal range. Enhancement was shown in 5/81 (6·2%) tests. The difference between inhibition pattern of normal leucocytes and CML remission leucocytes was statistically significant ($\chi^2 = 36·43$ at 1 d.f., $P < 0·001$). The difference between enhancement of migration shown by normal leucocytes and remission leucocytes was not statistically significant. However, it is thought that enhancement of migration is a phenomenon of weak sensitization (Cochran *et al.*, 1974). The number of positive reactions with normal leucocytes should therefore be considered as 23/81. Even then, the difference between positive reactions using remission leucocytes and normal leucocytes is highly significant ($\chi^2 = 27·29$ at 1 d.f., $P < 0·001$). Eliminating 9 autochthonous reactions from the total number of 57 tests carried out with CML remission leuco cytes (Table I), the difference between positive reactions using allogeneic remission leucocytes and normal leucocytes (inhibition + enhancement) is still significant ($\chi^2 = 23·53$ at 1 d.f., $P = < 0·005$).

Table 2 and the scattergram in Fig. 2 show the comparison of migration pattern of CML remission leucocytes in the presence of solubilized membrane antigens prepared from leukaemic cells and normal leucocytes. Fifteen out of 20 tests performed with normal leucocyte antigens showed migration of CML remission leucocytes within the normal range, while 2 showed enhancement and 3 showed inhibition of migration. The

TABLE II.—*Migration Pattern of Leucocytes Obtained from CML patients in Remission in Presence of Solubilized Membrane Antigens of Leukaemic Cells and Normal Leucocytes*

| Antigen donors | Migration pattern* Inhibition | Normal | Enhancement |
|---|---|---|---|
| CML leucocytes | 43/57† (75·4%) | 14/57‡ (24·6%) | Nil |
| Normal leucocytes | 3/20 (15%) | 15/20 (75%) | 2/20 (10%) |

* No. showing reactivity/No. tested.
† Eight of these represented autochthonous reactions.
‡ One of these represented autochthonous reaction.

frequency of enhancement, again, was not statistically significant. The difference between inhibition pattern with leukaemic and normal antigens was statistically significant ($\chi^2 = 19{\cdot}98$ at 1 d.f., $P < 0{\cdot}005$). Similarly the difference between positive reaction (enhancement and inhibition together) with leukaemic and normal antigens was significant ($\chi^2 = 13{\cdot}91$ at 1 d.f., $P < 0{\cdot}005$).

### DISCUSSION

Recently, leucocyte migration inhibition tests have been used to study tumour-specific cellular immunity in a variety of solid tumours (Anderson *et al.*, 1970; Mavligit *et al.*, 1972; Braun *et al.*, 1972; Cochran *et al.*, 1973*a*, 1974; and Kjaer, 1974). Santos *et al.* (1973) reported the *in vitro* production of MIF by remission leucocytes of acute leukaemic patients, which was tested indirectly on guinea-pig macrophages. Gutterman (1973*a*) used LMI test for studying cellular immunity in a few cases of leukaemias which included one CML in blast crisis.

The present report provides evidence for autochthonous, as well as allogeneic, sensitization of CML patients in remission to solubilized membrane antigens of leukaemic cells. The autochthonous reactivity was quite remarkable in that 8/9 tests showed inhibition of migration, and most of them showed a very high degree of inhibition (Fig. 1, circled points). In four cases where antigens prepared from relapse leukaemic cells were tested on autochthonous remission leucocytes, 6/7 antigenic samples inhibited leucocyte migration, indicating similarity or constancy of leukaemic antigens during serial relapses.

When leukaemic cell antigens were tested on allogeneic CML remission leucocytes, 35/49 (71·4%) tests showed inhibition of migration. It has been suggested before that use of soluble antigens can discriminate between tumour-specific and HL-A reactivity (Gutterman *et al.*, 1972*b*). However, recently, Dean *et al.* (1975) have reported positive blastogenesis of normal lymphocytes using solubilized tumour antigens. In our studies, the same batch of leukaemia antigens did not significantly inhibit the migration of normal leucocytes (22·2%). Similarly, soluble antigens prepared from normal leucocytes did not significantly inhibit migration of CML remission leucocytes (15%). These results, and the reports by Anderson *et al.* (1970) and Mavligit *et al.* (1972) using solid tumours, suggest that perhaps LMI is capable of distinguishing tumour-specific reactivity from the HL-A reactivity.

While studying tumour-specific sensitization with LMI test in breast cancer, Cochran *et al.* (1974) have noted enhancement of migration in a significant number of cases. This was suggested to be indicative of weak sensitization. In the present series of experiments enhancement to a significant level was not obtained with any of the antigen-leucocyte combination.

### REFERENCES

ANDERSON, V., BJERRUM, O., BENDIXEN, G., SCHIODT, T. & DISSING, I. (1970) Effect of Autologous Mammary Tumour Extracts on Human Leukocyte Migration *in vitro*. *Int. J. Cancer*, **5**, 357.

ANDERSON, P. N., KLEIN, D. L., BIAS, W. B., MULLINS, G. M., BURKE, P. J. & SANTOS, G. W. (1974) Cell-mediated Immunological Reactivity of Patients and Siblings to Blast Cells from Adult Acute Leukemias. *Israel J. Med. Sci.*, **10**, 1033.

BRAUN, M., SEN, L., BACHMANN, A. E. & PAVLOVSKY, A. (1972) Cell Migration Inhibition in Human Lymphomas Using Lymph Node and Cell Line Antigens. *Blood*, **39**, 368.

CHAR, D. H., LEPOURHIET, A., LEVENTHAL, B. G, & HERBERMAN, R. B. (1973) Cutaneous Delayed Hypersensitivity Responses to Tumour Associated and Other Antigens in Acute Leukemia. *Int. J. Cancer*, **12**, 409.

COCHRAN, A. J., JEHN, U. W. & GOTHOSKAR, B. (1973*a*) Immunity to Malignant Melanoma. In *Proceedings of VIIIth Inter. Pigment Cell Conference*, **1**, 360. Basle: Karger.

COCHRAN, A. J., KLEIN, G., KIESSLING, R. & GUNVEN, P. (1973*b*) Migration Inhibition Effect of Sera from Patients with Burkitt's Lymphoma. *J. natn. Cancer Inst.*, **51**, 1431.

COCHRAN, A. J., GRANT, R. M., SPILG, W. G., MACKIE, R. M., ROSS, C. E., HOYLE, D. E. & RUSSEL, J. M. (1974) Sensitization to Tumour Associated Antigens in Human Breast Carcinoma. *Int. J. Cancer*, **14**, 19.

DEAN, J. H., SILVA, J. S., MCCOY, J. L., LEONARD, C. M., MIDDLETON, M., CANNON, G. B. & HERBERMAN, R. B. (1975) Lymphocyte Blastogenesis Induced by Potassium Chloride Extracts of Allogeneic Breast Carcinoma and Lymphoid Cells. *J. natn. Cancer Inst.*, **54**, 1295.

FEDERLIN, K., MAINI, R. N., RUSSEL, A. S. & DUMONDE, D. C. (1971) A Micro-method for Peripheral Leukocyte Migration in Tuberculin Sensitivity. *J. clin. Path.*, **24**, 533.

FREEDMAN, W. H. & KOURILSKY, F. M. (1969) Stimulation of Lymphocytes by Autologous Leukemic Cells in Acute Leukemia. *Nature, Lond.*, **224**, 277.

GUTTERMAN, J. U., HERSH, E. M., KENNETH, B. M., MCCREDIE, R. B., BODEY (SR), G. P., RODRIGUEZ, V. & FREIREICH, E. J. (1972*a*) Lymphocyte Blastogenesis to Human Leukemia Cells and Their Relationship with Serum Factor, Immunocompetence and Prognosis. *Cancer Res.*, **32**, 2524.

GUTTERMAN, J. U., MAVLIGIT, G., MCCREDIE, K. B., BODEY, G. P., FREIREICH, E. J. & HERSH, E. M. (1972*b*) Antigen Solubilized from Human Leukemia: Lymphocyte Stimulation. *Science*, **177**, 1114.

GUTTERMAN, J. U. (1973*a*) In *Migration Inhibition Assays, Discussion. Natn. Cancer Inst. Monogr.*, **37**, 144.

GUTTERMAN, J. U., MAVLIGIT, G., MCCREDIE, K. B., FREIREICH, E. J. & HERSH, E. M. (1973*b*) Autoimmunization with Acute Leukemia Cells. Demonstration of Increased Lymphocyte Responsiveness. *Int. J. Cancer*, **11**, 521.

GUTTERMAN, J. U., ROSSEN, R. D., BUTLER, W. T., MCCREDIE, K. B., BODEY, G. P., FREIREICH, E. J. & HERSH, E. M. (1973*c*) Immunoglobulin on Tumour Cells and Tumour Induced Lymphocyte Blastogenesis in Human Acute Leukemia. *New Engl. J. Med.*, **288**, 169.

HALTERMAN, R. H. & LEVENTHAL, B. G. (1971) Enhanced Immune Response to Leukemia. *Lancet*, ii, 704.

KJAER, M. (1974) *In vitro* Demonstration of Cellular Hypersensitivity to Tumour Antigens by Means of Leukocyte Migration Technique in Patients with Renal Carcinoma. *Eur. J. Cancer*, **10**, 523.

LEVENTHAL, B. G., HALTERMAN, R. H., ROSENBERG, E. B. & HERBERMAN, R. B. (1972) Immune Reactivity of Leukemia Patients to Autologous Blast Cells. *Cancer Res.*, **32**, 1820.

LOWRY, O. H., ROSEBROUGH, N. J., FARR, A. L. & RANDALL, R. J. (1951) Protein Measurement with Folin Phenol Regaent. *J. biol. Chem.*, **193**, 265.

MAVLIGIT, G., GUTTERMAN, J. U., MCBRIDE, C. M. & HERSH, E. M. (1972) Multifaceted Evaluation of Human Tumour Immunity Using a Salt Extracted Colon Carcinoma Antigen. *Proc. Soc. exp. Biol. Med.*, **140**, 1240.

OREN, M. E. & HERBERMAN, R. B. (1971) Delayed Cutaneous Hypersensitivity Reactions to Membrane Extracts of Human Tumour Cells. *Clin. Exp. Immunol.*, **8**, 10.

POWLES, R. L., BALCHIN, L. A., FAIRLEY, G. H. & ALEXANDER, P. (1971) Recognition of Leukemic Cells as Foreign before and after Autoimmunization. *Br. med. J.*, i, 486.

ROSENBERG, E. B., HERBERMAN, R. B., LEVINE, R. H., HALTERMAN, R. H., MCCOY, J. L. & WUNDERLICH, J. R. (1972) Lymphocyte Cytotoxicity Reactions to Leukemia Associated Antigens in Identical Twins. *Int. J. Cancer*, **9**, 648.

SANTOS, G. W., MULLINS, G. M., BIAS, W. B., ANDERSON, P. N., GRAZIANO, K. D., KLEIN, D. L. & BURKE, P. J. (1973) Immunologic Studies in Acute Leukemia. *Natn. Cancer Inst. Monogr.*, **37**, 69.

VIZA, D. C., BERNARD-DEGANI, O., BERNARD, C. L. & HARRIS, R. (1969) Leukemia Antigens. *Lancet*, ii, 493.